# Comprehensive transcriptome analysis reveals heat-responsive genes in flowering Chinese cabbage (*Brassica campestris* L. ssp. *chinensis*) using RNA sequencing

**DOI:** 10.3389/fpls.2022.1077920

**Published:** 2022-12-02

**Authors:** Muhammad Ikram, Jingfang Chen, Yanshi Xia, Ronghua Li, Kadambot H. M. Siddique, Peiguo Guo

**Affiliations:** ^1^ Guangdong Provincial Key Laboratory of Plant Adaptation and Molecular Design, International Crop Research Center for Stress Resistance, School of Life Sciences, Guangzhou University, Guangzhou, China; ^2^ The UWA Institute of Agriculture, UWA School of Agriculture & Environment, The University of Western Australia, Perth, WA, Australia

**Keywords:** heat stress, transcriptome, differentially expressed genes, cluster analysis, flowering chinese cabbage, RT-qPCR

## Abstract

Flowering Chinese cabbage (*Brassica campestris* L. ssp. *chinensis* var. *utilis* Tsen et Lee, 2n=20, AA) is a vegetable species in southern parts of China that faces high temperatures in the summer and winter seasons. While heat stress adversely impacts plant productivity and survival, the underlying molecular and biochemical causes are poorly understood. This study investigated the gene expression profiles of heat-sensitive (HS) ‘3T-6’ and heat-tolerant (HT) ‘Youlu-501’ varieties of flowering Chinese cabbage in response to heat stress using RNA sequencing. Among the 37,958 genes expressed in leaves, 20,680 were differentially expressed genes (DEGs) at 1, 6, and 12 h, with 1,078 simultaneously expressed at all time points in both varieties. Hierarchical clustering analysis identified three clusters comprising 1,958, 556, and 591 down-regulated, up-regulated, and up- and/or down-regulated DEGs (3205 DEGs; 8.44%), which were significantly enriched in MAPK signaling, plant–pathogen interactions, plant hormone signal transduction, and brassinosteroid biosynthesis pathways and involved in stimulus, stress, growth, reproductive, and defense responses. Transcription factors, including MYB (12), NAC (13), WRKY (11), ERF (31), HSF (17), bHLH (16), and regulatory proteins such as PAL, CYP450, and photosystem II, played an essential role as effectors of homeostasis, kinases/phosphatases, and photosynthesis. Among 3205 DEGs, many previously reported genes underlying heat stress were also identified, e.g., *BraWRKY25, BraHSP70, BraHSPB27, BraCYP71A23, BraPYL9*, and *BraA05g032350.3C*. The genome-wide comparison of HS and HT provides a solid foundation for understanding the molecular mechanisms of heat tolerance in flowering Chinese cabbage.

## Introduction

Heat stress is an environmental factor affecting crop vegetative growth, performance, and productivity worldwide, even causing plant death (Caers et al., 1985; Song et al., 2014a). According to a crop-based algorithm study, a 1°C rise in seasonal temperature causes 2.5–16% direct yield losses for key crops in tropical and subtropical regions (Lobell et al., 2008). Furthermore, greenhouse gas emissions have increased atmospheric temperatures by 0.3°C per decade (Jones et al., 1999), indicating that extreme temperature and climate change events will threaten food security by decreasing crop production (Battisti and Naylor, 2009; Rosenzweig et al., 2014). Flowering Chinese cabbage (*Brassica campestris* L. ssp. *chinensis* var. *utilis* Tsen et Lee) is a commercial vegetable crop mainly cultivated in southern parts of China (Chen et al., 2017). The demand for flowering Chinese cabbage is increasing due to its rich source of vitamin C, favourable taste, soluble fiber, phenolics, glucosinolates, and other nutritional value (Huang et al., 2017), but southern China’s high summer and autumn temperatures seriously impair crop quality and production (Fan et al., 2017). Heat stress adversely affects plant growth, phenological stages, grain filling, pollen viability, even the structural changes in tissues and cell organelles, loss of leaf water, cell membrane damage, and photosynthetic membranes (Yoshinaga et al., 2005). At a molecular level, heat stress also affects the synthesis of primary and secondary metabolites, antioxidant enzymes, and lipid peroxidation *via* the production of reactive oxygen species (ROS). However, plants have evolved various molecular and physiological mechanisms during domestication to resist heat stress (Wahid et al., 2007; Hasanuzzaman et al., 2013), such as accumulating different metabolites (antioxidants, osmoprotectants, etc.) and activation of signaling and metabolic pathways. Therefore, understanding the mechanism of signaling cascades and specific genes expressed in response to HT will be beneficial for developing stress-tolerant varieties.

Transcription factors (TFs), signal transduction components, and proteins related to metabolism are responsive to heat stress (Grover et al., 2013). Recently, heat-responsive siRNAs and miRNAs in flowering Chinese cabbage have been reported (Yu et al., 2012; Song et al., 2021), with most up-regulated under high temperature, and their target genes with differential gene expression involved in responses to temperature stimulus, cell membrane, signal transduction, and mitogen-activated protein kinase (MAPK) signaling pathways (Song et al., 2021). In Chinese cabbage, Song et al. (2016) used RNA-seq datasets of heat treatments to identify 14,329 DEGs and 9,687 novel LncRNAs, of which LncRNAs controlled 192 DEGs under heat stress. Transcriptome sequencing analysis of Chinese cabbage (*B. rapa* ssp. *pekinensis*) inbred lines revealed that heat stress affects many genes, including those linked with membrane leakage, heat-shock proteins (HSPs), and enzymes that regulate ROS homeostasis (Dong et al., 2015). Heat-shock transcriptional factors (HSFs), which stimulate the expression of HSPs and other stress proteins, play a critical role in modulating stress responses (Swindell et al., 2007; Dong et al., 2015; Liu et al., 2018; Yao et al., 2020). The HSF gene family has been identified in various species, including *Arabidopsis* (Nover et al., 2001), tomato (Yang et al., 2016), soybean (Li et al., 2014), potato (Dossa et al., 2016), and Chinese cabbage (Song et al., 2014b). HSF and HSP family members are involved in several stress response pathways, including heat, cold, osmotic, and salt, in *Arabidopsis* (Swindell et al., 2007), with 21 HSFs cloned and analyzed (Nover et al., 2001). In global transcription profiles, many HSFs/HSPs were up-regulated in *Brassica napus* siliques, with many other heat-responsive marker genes, such as *ROF2, MBF1c, DREB2a*, and *Hsa32*, involved in heat resistance in many plants (Yu et al., 2014). Another study found that genes involved in protein protection, biotic stress responses, oxidative stress, programmed cell death, and metabolism were differentially expressed during heat stress (Kotak et al., 2007). Only limited transcriptome data are available for flowering Chinese cabbage under heat stress relative to Chinese cabbage, maize, rice, and Arabidopsis (Song et al., 2014a; Chen et al., 2017; Fan et al., 2017; Huang et al., 2017; Ali et al., 2022).

In recent years, next-generation sequencing technologies have been developed and significantly reduced the cost and increased the efficiency of genome sequencing. Therefore, the entire genome of *B. rapa* (Chiifu-401-42) was sequenced using Illumina GA II sequencing and annotated (Wang et al., 2011), which provides the foundation to identify the heat-responsive genes in flowering Chinese cabbage. Using RNA-seq, researchers can identify the expressed genes, particularly those with low abundance. To date, microarrays and RNA-Seq projects have been undertaken in many species, including rice, Chinese cabbage, maize, cotton, Arabidopsis, and vegetables, to detect genes responsive to heat and cold stress (Grover et al., 2013; Nakashima et al., 2014; Ikram et al., 2020; Song et al., 2021; Ali et al., 2022). For example, in *Arabidopsis*, nearly 30% of DEGs associated with abiotic stresses were identified, and 2,409 genes were involved in salt, cold, and drought stresses (Kreps et al., 2002). In wheat, 2% of genes were found to be associated with cold stress (Winfield et al., 2010). In *A. mongolicus*, 9,309 and 23,419 up- and down-regulated genes were detected under cold stress (Pang et al., 2013). In previous studies, various genes have been functionally characterized for heat tolerance in different crop plants, such as *ZmWRKY106* (Wang et al., 2018), *PpEXP1* (Xu et al., 2014), *hsp26* (Xue et al., 2010), *WsSGTL1* (Mishra et al., 2013), and *OsOr-R115H* (Jung et al., 2021).

Our previous research identified expressed sequence tag-simple sequence repeat (EST-SSR) markers in flowering Chinese cabbage (Chen et al., 2017) and also detected significant SNPs/genes for plant height (Ikram et al., 2022a; Ikram et al., 2022b; Li et al., 2022) and wilt resistance (Lai et al., 2021) in tobacco. The present study used RNA-seq based on the Illumina HiSeq2000 platform to undertake a genome-wide analysis of gene expression in leaves of HT (Youlu-501) and HS (3T-6) flowering Chinese cabbage varieties under heat stress (0, 1, 6, and 12 h) to (1) identify DEGs in HS and HT genotypes after 1, 6, and 12 h of heat stress: (2) annotate these genes based on gene ontology (GO) and Kyoto Encyclopedia of Genes and Genomes (KEGG) enrichment analysis; (3) identify the expression pattern of genes using Hierarchical clustering analysis; (4) identify TFs associated with the DEGs and their role in heat stress. The findings of this study will provide new insights into the heat tolerance of flowering Chinese cabbage.

## Materials and methods

### Plant materials and physiological parameters

Two natural flowering Chinese cabbage (*Brassica campestris L. ssp. chinensis* var. *utilis Tsen et Lee*) varieties [3T-6 (heat-sensitive) and Youlu-501 (heat-tolerant)] were obtained from the Guangdong Academy of Agricultural Sciences, China. The seeds of both accessions were sterilized and then germinated in a growth chamber of our lab at the International Crop Research Center for Stress Resistance (113.37_E, 23.05_N), Guangzhou University, under the following condition: 28/22°C for 14/10 h (day/night) with 80% relative humidity. Fifteen-day-old seedlings at five-leaf stage were transferred into another chamber at 38/29°C (14/10 h day/night) for heat stress. After the heat-stress treatment, samples were collected at three time points from fully expanded upper leaves of heat-sensitive (HS-1h, HS-6h, and HS-12h) and heat-tolerant (HT-1h, HT-6h, and HT-12h) plants. For the control (CK), leaf samples were collected from HS (HS-CK) and HT (HT-CK) plants under normal conditions at 25°C. The harvested leaf samples were immediately frozen in liquid nitrogen and preserved at –80°C until RNA extraction. The experiment had five replicates. Peroxidase (POD), superoxide dismutase (SOD), and catalase (CAT) were assessed as described in previous studies (Kono, 1978; Aebi, 1984). The fresh plants were taken at different times to calculate the fresh weight in both varieties.

### RNA extraction, cDNA library construction, and sequencing

For transcriptome assembly, total RNA was extracted from five biological replicates at CK, HS/HT-1h, HS/HT-6h, and HS/HT-12h using TRIzol reagent (Takara Bio, Ostu, Japan), following the manufacturer’s recommendations. The RNA was pooled into a single sample in equal amounts to find the broad gene library associated with heat resistance. DNase I (Takara Bio, Ostu, Japan) was used to digest genomic DNA, and RNA integrity and quantity were assessed using 1% agarose gel electrophoresis and a microplate spectrophotometer (BioTek Company, USA). cDNA libraries were prepared using RNA with high purity. In brief, mRNAs were purified using poly-T oligo-attached magnetic beads from total RNA. Following fragmentation, first-strand cDNA was synthesized using a random hexamer primer, followed by second-strand cDNA synthesized using DNA Polymerase I and RNase H. The paired-end Illumina sequencing required the purification and ligation of the double-stranded cDNAs to adaptors. After PCR amplification, library quality was verified on the Agilent 2100 Bioanalyzer system before sequencing the cDNA libraries using the Illumina HiSeq2000 system by the Beijing Genomics Institute (BGI) to generate 125 bp paired-end reads.

### RNA-seq data analysis and annotation

Before library assembly, the raw reads were processed using SOAPnuke v1.5.2 (https://github.com/BGI-flexlab/SOAPnuke; Cock et al., 2009) to remove low-quality reads with >50% of bases with Q ≤ 20 or >10% unknown (N) bases and adapter sequences. The raw reads with quality check parameters were transformed to clean reads for further analysis. Finally, clean reads were mapped and aligned to *B. rapa* cv. Chiifu v3.0 reference genome (http://brassicadb.org/brad/; Wang et al., 2011) using HISAT2 v2.1.0 (Kim et al., 2015) and Bowtie2 v2.2.5 (Langmead and Salzberg, 2012). For functional annotation, all assembled gene sequences were aligned to databases, including reference genome (BRAD database), NT, NR, UniProt, Cluster of Orthologous Groups of proteins (COG), KEGG, and GO using Trinotate software (http://trinotate.github.io/) with E value ≤1e-5. The mapped reads were used to measure expression levels in fragments per kilobase of exon per million mapped fragments (FPKM) using RSEM software (Li and Dewey, 2011). Pearson’s correlation coefficients for the eight samples were estimated using the ‘cor’ function in R4.1.0 (http://www.r-project.org/) software based on FPKM values, and a correlation graph was drawn using the ‘ggplot2’ R package (https://cran.r-project.org/web/packages/ggplot2/index.html). The ‘prcomp’ function in R4.1.0 (http://www.r-project.org/) was used to perform principal component analysis (PCA).

### Differential expression and cluster analyses

For HS and HT varieties, DEGs between control and heat-stress conditions were calculated using the DESeq2 R package (Love et al., 2014), a reliable and efficient package for detecting DEGs between diverse samples. The significant DEGs between heat stress and control conditions were identified at |log_2_(fold-change) | > 2 and false discovery rate (FDR) adjusted *P* < 0.001. Further, significant DEGs in HS or HT were exposed to hierarchical or K-mean clustering based on the hclust function in R4.1.0 (distance: euclidean; method: ward.d), with log_2_FC subjected as the input. Finally, DEGs were analyzed for soft clustering based on fuzzy c-mean using the R4.1.0 package ‘Mfuzz’ (Kumar and Futschik, 2007).

### Gene ontology and KEGG pathway enrichment analysis of differentially expressed genes

GO and KEGG enrichment analysis was undertaken to reveal the functional annotation and pathways associated with up- and down-regulated DEGs in each cluster or group. An online tool (http://www.geneontology.org/) was used to conduct GO enrichment analysis at FDR adjusted *P* ≤ 0.05, classifying the DEG functions into three categories: cellular component, biological process, and molecular function. Similarly, KEGG pathway enrichment analysis was performed using the KEGG database (https://www.genome.jp/kegg/pathway.html) with significant criteria at an adjusted *P* ≤ 0.05 (Kanehisa and Goto, 2000).

### Validation of RNA-seq result by real-time quantitative PCR

Ten genes were chosen randomly to confirm the reliability of RNA-seq results using RT-qPCR. The RNA was the same as that used for transcriptomic sequencing, with the relative expression calculated using ABIPrism7000 RT-qPCR platform (Applied Biosystems, USA). The PCR reaction volume was 10 mL with 1 μg cDNA, 5 mL SYBR Premix Ex Taq II (Takara, China), and 200 nM primers. Three technical and biological replicates for each sample were measured with the following protocol: 95°C for 5 min, followed by 40 cycles of 95°C for 15 s, 60°C for 60 s, and until 65°C to 97°C with a ramp rate of 0.02°C s^–1^ for dissociation curve analysis. *BraActin* was used as housekeeping gene to normalize the data. The relative expression level of all selected genes at each time point was calculated using the 2^−ΔΔCT^ method. The in-house R script was used to conduct a student t-test at P ≤ 0.05 to find the significant differences between time points.

## Results

### Plant phenotypic and enzymatic activities under heat stress

**Figure 1:**
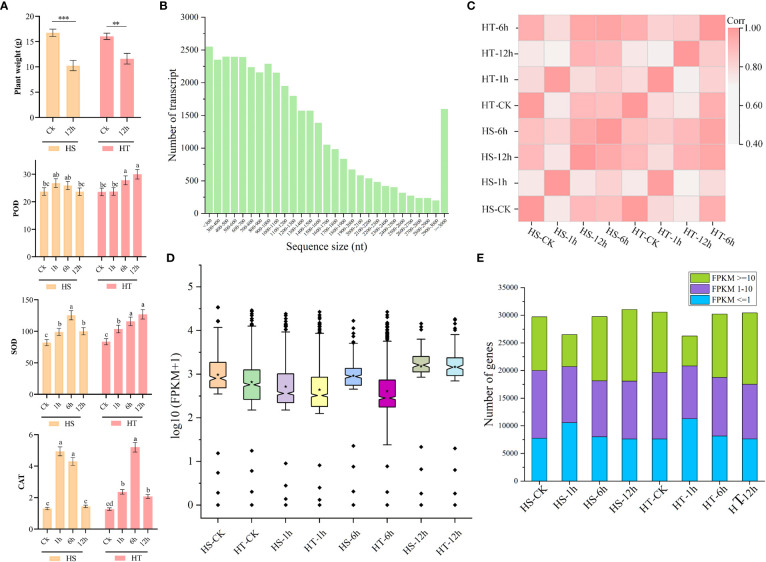
Phenotypic effect and overview of RNA sequencing data based on heat-sensitive and heat-tolerant flowering Chinese cabbage varieties. **(A)** Plant weight and enzymatic activities between control and stress conditions; **(B)** Number of transcripts and their sequence size; **(C)** Correlation analysis between heat-sensitive and heat-tolerant varieties at different time points; **(D)** log_10_ transformation of FPKM values at 0, 1, 6, and 12 h; **(E)** Distribution of genes based on FPKM values. The different small letters indicate the significant differences between the time points.

Heat stress significantly altered plant growth and the reproduction system. At the initial stage of stress, plant leaves exhibited moderate to high wilting symptoms. Twelve hours of heat stress significantly decreased whole plant weight (g) in both species relative to the control (**Figure 1A**). The heat treatment significantly increased catalase (CAT), superoxide dismutase (SOD), and peroxidase (POD) activities in plant leaves at all time points, relative to the control, and more so in Youlu-501 than 3T-6 (**Figure 1A**).

### Overview of transcriptomic sequencing data

Libraries were constructed and sequenced on the IlluminaHiseq2000 deep sequencing platform using the PE125 protocol (**Table S1**). Each library generated 21.94–24.90 million raw reads. After removing joint contamination, low-quality reads, and reads with unknown base (N) content, 21.86–24.81 million clean reads (99.42–99.963%) were collected (**Table S1**). The clean Q30 base rates of the eight samples ranged from 89.71–91.04%. Of the clean reads, 88.59–92.06% total and 73.06–76.72% unique reads were mapped to the *B. rapa* reference genome (**Table S1**), with 46,250 predicted *B. rapa* genes (**Figure 1B**). The PCA revealed an overall 86.80% (PC1 = 73.40% and PC2 = 13.40%) variation in gene expression datasets, with the heat-stress conditions (HS-1h/HR-1h, HS-6h/HR-6h, and HS-12/HR-12) clustered or grouped nearby but separated from their corresponding control HS-CK/HR-CK (**Figure S1**). Pearson’s correlation coefficient analysis based on FPKM values of each sample revealed a significant positive correlation between different time points; for example, HS-6h and HT-6h highly correlated with other time points compared to HS-12h and HR-12h (**Figure 1C**). The HS and HT varieties significantly differed based on the log_10_ transformation of FPKM (**Figure 1D**), with the FPKM values of most genes >10 (**Figure 1E**). Sixteen genes had FPKM values >2,000 in both varieties at all time points.

### Differential expression profiling of two varieties under heat stress

**Figure 2:**
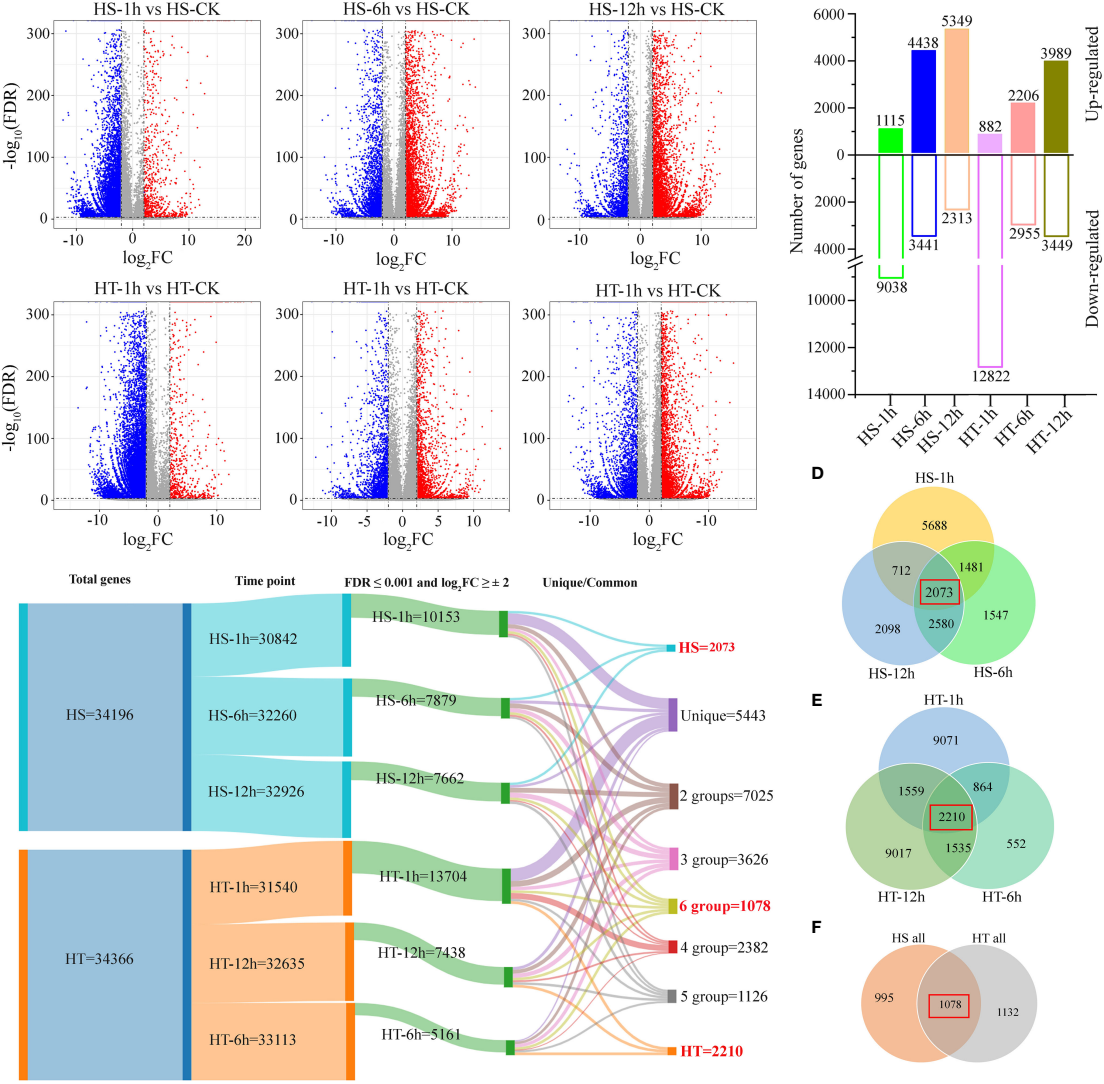
Analysis of differentially expressed genes in flowering Chinese cabbage varieties in response to heat stress. **(A)** Volcano plots of all expressed genes in heat-sensitive (HS) and heat-tolerant (HT) varieties at different time points relative to CK, with log_2_FC values drawn against –log_10_ (FDR) adjusted *P-*values. Red and blue dots represent up- and down-regulated DEGs based on |log_2_(foldchange)|≥2 and FDR ≤ 0.001; gray dots represent non-significant DEGs; **(B)** The Sankey diagram represents the number of common and unique DEGs between each stress point; **(C)** Number of up and down-regulated DEGs identified under different heat-stress treatments relative to control HS and HT varieties; **(D)** Common and unique DEGs under different heat-stress treatments in HS variety; **(E)** Common and unique DEGs in HT under different heat-stress treatments; **(F)** Common DEGs in all heat-stress treatments in HS and HT.

DESeq_2_ software was used to investigate DEGs with FPKM values >1. During heat stress, 30,842, 32,260, 32,926, 31,540, 32,635, and 33,113 genes were regulated for HS-1h, HS-6h, HS-12h, HT-1h, HT-6h, and HT-12h, respectively (**Figures 2A, B**). The DEGs were selected using the following criteria of log_2_ fold-change |log_2_(foldchange)|≥2 and FDR adjusted *P* ≤ 0.001. Volcano plots identified the significant up- or down-regulated genes during heat stress at each time point (**Figure 2A**), with 10,153 (1,115 up- and 9,038 down-regulated), 7,879 (4,438 up- and 3,441 down-regulated), and 7,662 (5,349 up- and 2,313 down-regulated) DEGs in 3T-6 at HS-1h, HS-6h, and HS-12h compared to the control (**Figures 2A–C**) and 13,704 (882 up- and 12,822 down-regulated), 5,161 (2,206 up- and 2,955 down-regulated), and 7,438 (3,989 up- and 3,449 down-regulated) DEGs in Youlu-501 at HT-1h, HT-6h, and HT-12h compared to the control (**Figures 2A–C**).

Subsequently, Sankey and Venn diagrams demonstrated the number of common and uniquely expressed genes at each time point or between two consecutive time points (**Figures 2B, D–F**), with 5,443 DEGs unique in all samples, and 7,025, 3,626, 2,382, 1,126, and 1,078 DEGs shared between two, three, four, five, and six samples (**Figure 2B**). Additionally, 2,073 and 2,210 DEGs were common at all time points of HS and HT genotypes, respectively (**Figures 2D, E** **)**, indicating higher number of genes were regulated in heat-tolerant variety than sensitive. Of these, 1,078 DEGs were co-expressed differently in the HS and HT genotypes (**Figure 2F**), with 237 and 747 up- and down-regulated, respectively, and 94 differentially regulated. After 6 and 12 h of heat treatment, 328/325 and 330/323 more DEGs were up-regulated in HS/HT. Finally, 3,205 genes were differentially expressed at 1, 6, and 12 h in 3T-6 or Youlu-501 (**Figures 2B, D–F**).

### Functional annotation of DEGs responsive to heat stress

**Figure 3:**
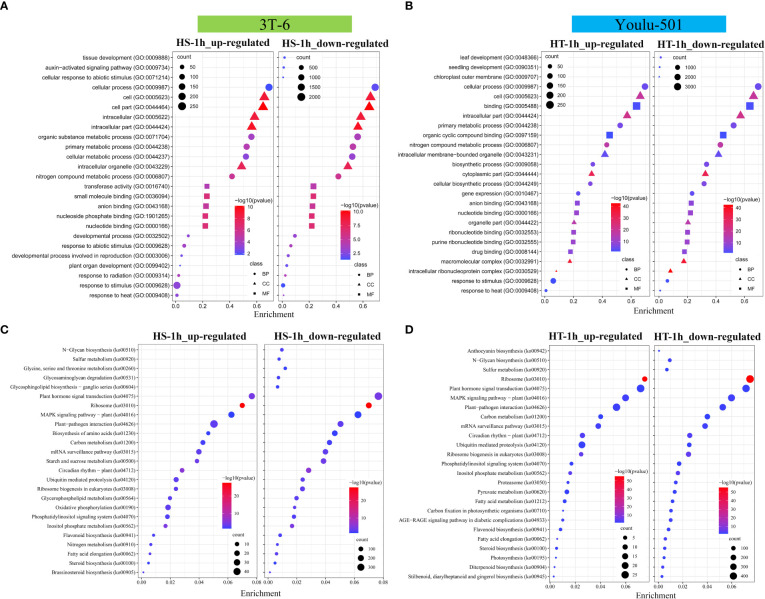
GO and KEGG enrichment analysis of up- and down-regulated genes in heat-sensitive (3T-6) and heat-tolerant (Youlu-501) flowering Chinese cabbage. **(A, B)** The top 25 GO terms in three categories are listed at HS-1h vs. HS-CK and HT-1h vs. HT-CK at *P* ≤ 0.05; **(C, D)** The top 25 KEGG pathways and their corresponding number of genes HS-1h vs. HS-CK and HT-1h vs. HT-CK at *P* ≤ 0.05. The x-axis represents the enrichment factor, the y-axis represents GO/KEGG terms with the number of genes, and the blue to red color indicates the significance and number of genes.

GO enrichment analyses of up- and down-regulated genes in HS and HT at 1, 6, and 12 h categorized the heat stress-related genes associated with key biological processes. **Figures 3**, **S2**–**S5** show the top 25 GO terms in three categories. In HS, for HS-CK vs. HS-1h, 462 up-regulated genes were mainly involved in ‘response to stimulus (GO:0009628),’ ‘cellular process (GO:0009987),’ ‘primary metabolic process (GO:0044238),’ ‘response to abiotic stimulus (GO:0009628),’ ‘developmental process involved in reproduction (GO:0003006),’ and ‘response to heat (GO:0009408)’ (**Figure 3A**). In comparison, most of the 4,055 down-regulated genes were involved in ‘tissue development (GO:0009888),’ ‘auxin-activated signaling pathway (GO:0009734),’ ‘cell part (GO:0044464),’ ‘intracellular part (GO:0044424),’ ‘cellular response to abiotic stimulus (GO:0071214),’ ‘cell (GO:0005623),’ ‘intracellular (GO:0005622),’ and ‘cellular process (GO:0009987)’ (**Figure 3A**). For HS-CK vs. HS-6h and HS-CK vs. HS-12h, 2,046 and 2,275 up-regulated genes were significantly enriched in ‘anion binding (GO:0043168),’ ‘nucleotide binding (GO:0000166),’ ‘response to temperature stimulus (GO:0009266),’ ‘purine nucleotide binding (GO:0017076),’ ‘carbohydrate derivative binding (GO:0097367),’ ‘response to abiotic stimulus (GO:0009628),’ and ‘photosynthesis (GO:0015979)’ (**Figure S2**). In contrast, 1,568 and 902 down-regulated genes were mainly enriched in ‘metabolic process (GO:0008152),’ ‘detoxification (GO:0071722),’ ‘intracellular organelle (GO:0043229),’ ‘chloroplast (GO:0009507),’ and ‘RNA binding (GO:0003723)’ (**Figure S2**). In HT, for HT-CK vs. HT-1h, 418 up-regulated genes were involved in ‘binding (GO:0005488)’ ‘cellular process (GO:0009987),’ and ‘primary metabolic process (GO:0044238)’, and 6,626 down-regulated genes were enriched in ‘leaf development (GO:0048366),’ ‘response to stimulus (GO:0009628),’ ‘chloroplast outer membrane (GO:0009707),’ and ‘biosynthetic process (GO:0009058)’ (**Figure 3B**). Similarly, the highest number of DEGs for HT-6h and HT-12 were involved in ‘response to stimulus (GO:0009628),’ ‘chloroplast (GO:0009507),’ ‘cell periphery (GO:0071944),’ ‘auxin homeostasis (GO:0010252),’ ‘membrane-bounded organelle (GO:0043227),’ and ‘photosynthesis (GO:0015979)’ (**Figure S3**). The GO enrichment analysis indicated that HT had more enriched DEGs for each term than HS; for example, 1,410 HT and 415 HS genes were involved in ‘response to stimulus’ (**Figures 3A, B**, **S2**, **S3**).

The KEGG database was used to investigate the key genes involved in KEGG pathways, with 2,389 and 1,453 up-regulated and 3,390 and 4,286 down-regulated genes in HS and HT, respectively, enriched in 39 KEGG pathways (adjusted *P* ≤ 0.05), including ‘plant-pathogen interaction (ko04626),’ ‘oxidative phosphorylation (ko00190),’ ‘sulfur metabolism (ko00920),’ ‘steroid biosynthesis (ko00100),’ ‘nitrogen metabolism (ko00910),’ ‘plant hormone signal transduction (ko04075),’ ‘brassinosteroid biosynthesis (ko00905),’ ‘MAPK signaling pathway - plant (ko04016),’ ‘circadian rhythm - plant (ko04712),’ ‘carbon metabolism (ko01200),’ and ‘photosynthesis (ko00195)’ (**Figures 3C, D**, **S4**, **S5**). The HT genes were more involved in signal transduction and MAPK pathways than the HS genes (**Figures 3C, D**).

### Cluster analysis of differentially expressed genes

**Figure 4:**
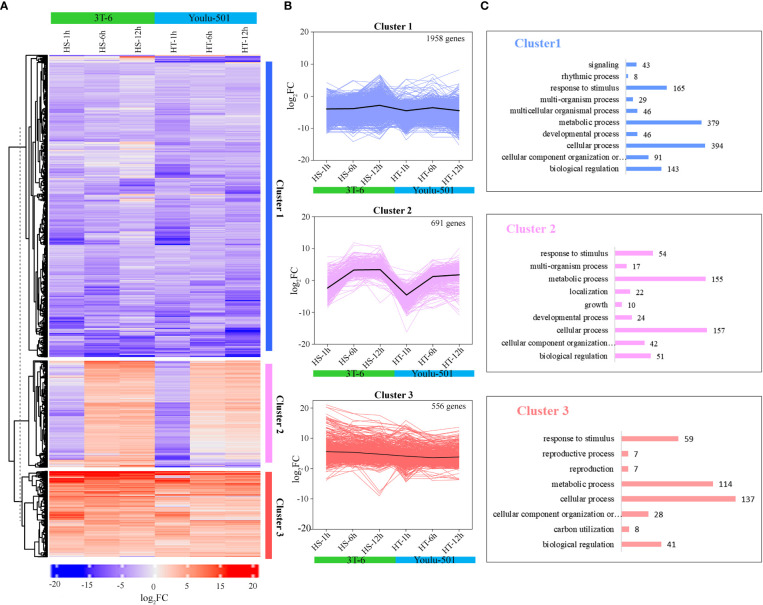
Expression patterns of differentially expressed genes (DEGs) and their functional annotation in heat-sensitive (3T-6) and heat-tolerant (Youlu-501) flowering Chinese cabbage in response to heat stress. **(A)** Hierarchical clustering analysis indicates DEG expression patterns in 3T-6 and Youlu-501 after 1, 6, and 12 h of heat stress relative to their controls; **(B)** Cluster analysis shows that the lines reflect expression patterns for each DEG at heat-stress time points. Black lines in each cluster indicate mean changes in DEG expression; **(C)** Gene ontology enrichment analysis of DEGs in each cluster.

We used gene expression changes (log_2_FC value) to perform hierarchical clustering for 3205 DEGs (**Figures 2D–F**) in response to heat stress among flowering Chinese cabbage varieties. The clusters with similar patterns of expression change were identified using the hclust method. As a result, we found three clusters of genes response to heat stress at three time points relative to control and a black line representing the mean changes in expression (**Figures 4A–C** and **Table S2**). Cluster 1 contained 1,958 DEGs that were down-regulated in both varieties at 1, 6, and 12 h, and the expression variations of cluster 1 genes in Youlu-501 were comparatively more repressed than in 3T-6 (**Figures 4A, B** **;** **Table S2**). Interestingly, cluster 2 included 691 DEGs, the expression changes were down-regulated at 1h in both varieties compared to the control and up-regulated at 6 and 12 h, but expression patterns of HT were relatively higher than HS at 6 and 12 h stress condition **(** **Figures 4A, B** **;** **Table S2**). A total of 556 DEGs were found in Cluster 3, and most of them were up-regulated by heat stress in both varieties. The difference in Cluster 3 was that the genes had moderately greater induced expression levels in HS than in HT during heat stress except for 12h (**Figures 4A, B** **;** **Table S2**).

Further, we investigated the biological functions of these clusters using GO enrichment analysis (**Figure 4C** **;** **Table S2**). The cluster 1 DEGs were mainly involved in cellular component organization or biogenesis (91), biological regulation (143), cellular process (394), developmental process (46), metabolic process (379), and response to stimulus (165). The DEGs belonging to developmental process (24), growth (10), response to stimulus (54), localization (22), metabolic process (155), and multi-organism process (17) were also significantly enriched in cluster 2. In addition, DEGs in cluster 3, which were up-regulated in both varieties, were involved in biological regulation (41), carbon utilization (8), biogenesis (28), reproduction (7), and response to stimulus (59). The fold-change expression values were higher in HT Youlu-501 than in HS 3T-6 (**Figure 4A** **;** **Table S2**). In brief, the top 15 potential DEGs expressed in all stages were identified, and their homologous have been reported to play an essential role in heat stress (**Table 1**).

**Table 1:** List of top potential differentially expressed genes involved in heat tolerance in flowering Chinese cabbage.

Clusters	Gene id	Log_2_ fold-change						Functional annotation	
		HS-1h	HS-6h	HS-12h	HT-1h	HT-6h	HT-12h	Annotation	GO/KEGG pathways enrichment
Cluster3	*BraA07g024750.3C*	9.07	4.65	3.32	7.49	2.69	3.43	Abscisic acid receptor PYL9-like	MAPK signaling pathway - plant (ko04016); Plant hormone signal transduction (ko04075)
Cluster1	*BraA09g028180.3C*	–3.92	–4.92	–6.18	–3.67	–6.00	–7.19	Sodium transporter HKT1-like	
Cluster1	*BraA09g022320.3C*	–4.50	–3.13	–2.66	–4.92	–2.55	3.01	abscisic acid receptor PYR1-like	MAPK signaling pathway - plant (ko04016); Plant hormone signal transduction (ko04075)
Cluster1	*BraA09g047180.3C*	-4.96	-6.50	-2.38	-4.78	-2.22	-3.83	stomatal closure-related actin-binding protein 3	microspherule protein 1 (K11674); cytoskeletal protein binding (GO:0008092)
Cluster3	*BraA10g001200.3C*	3.67	7.01	3.98	-2.12	2.20	2.67	gibberellin 2-beta-dioxygenase 6	Biosynthesis of secondary metabolites (ko01110); oxidoreductase activity (GO:0016491); metal ion binding (GO:0046872)
Cluster2	*BraA10g011520.3C*	-2.31	2.21	3.13	-3.24	2.12	3.06	abscisic acid receptor PYL8-like	MAPK signaling pathway - plant (ko04016); Plant hormone signal transduction (ko04075)
Cluster3	*BraA10g016090.3C*	3.12	5.68	5.87	2.90	3.98	4.63	temperature-induced lipocalin-1-like	apolipoprotein D and lipocalin family protein (K03098); transporter activity (GO:0005215)
Cluster3	*BraA10g028350.3C*	4.58	6.06	4.35	4.03	5.64	4.56	abscisic acid receptor PYL5	MAPK signaling pathway - plant (ko04016); Plant hormone signal transduction (ko04075)
Cluster3	*BraA02g041550.3C*	5.16	4.93	4.36	3.58	3.35	4.59	E3 ubiquitin-protein ligase MIEL1	Ubiquitin mediated proteolysis (ko04120); cation binding (GO:0043169); metal ion binding (GO:0046872)
Cluster1	*BraA05g008340.3C*	-3.79	-8.87	-8.87	-3.12	-8.79	-8.79	indole-3-acetic acid-induced protein ARG7-like	Plant hormone signal transduction (ko04075); response to stimulus (GO:0050896); response to hormone (GO:0009725); response to auxin (GO:0009733)
Cluster1	*BraA06g037940.3C*	-7.17	-10.75	-5.54	-4.92	-3.45	-7.65	pollen-specific protein-like At4g18596	
Cluster1	*BraA07g002070.3C*	-5.62	-5.62	-4.04	-5.09	-3.60	-4.24	putative RING-H2 finger protein ATL49	E3 ubiquitin-protein ligase RNF38/44 [EC:2.3.2.27] (K19041); intrinsic component of membrane (GO:0031224)
Cluster3	*BraA05g042010.3C*	8.06	5.70	8.80	5.97	3.29	4.39	protein DETOXIFICATION 24-like	multidrug resistance protein (K03327); antiporter activity (GO:0015297); transporter activity (GO:0005215)
Cluster1	*BraA08g000300.3C*	-3.10	-4.78	-3.14	-2.98	-4.63	-3.50	cellulose synthase-like protein E1	cellulose synthase A [EC:2.4.1.12] (K10999)
Cluster1	*BraA05g032350.3C*	-3.28	-2.36	-5.58	-2.30	-4.73	-3.55	bidirectional sugar transporter SWEET2-like	solute carrier family 50 (K15382); cell wall organization or biogenesis (GO:0071554); organic substance biosynthetic process (GO:1901576)

HS, heat-sensitive; HT, heat-tolerant.

### Gene families associated with heat stress

**Figure 5:**
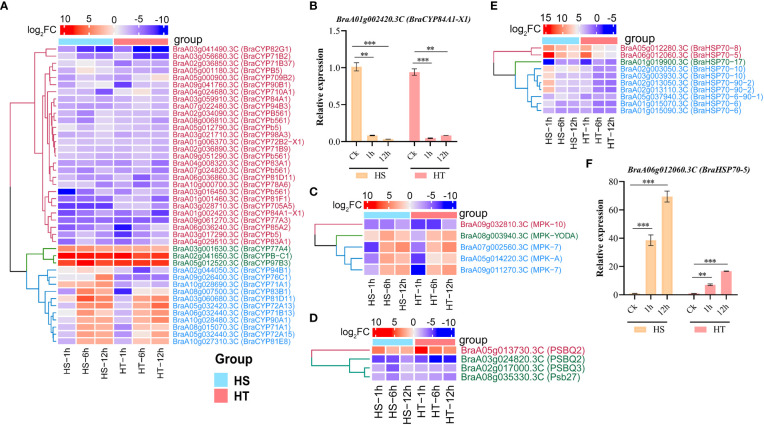
Heatmaps of up- and down-regulated genes in heat-sensitive (3T-6) and heat-tolerant (Youlu-501) Chinese flowering cabbage related to stress at different times and confirmation of two DEGs through RT-qPCR analysis. **(A)** Number of DEGs of cytochrome P450 gene family at four heat-stress time points; **(B)** *BraA01g002420.3C* (*BraCYP84A1-X1* cytochrome P450 84A1 isoform X1) showing similar expression pattern in RNA-seq data; Heatmaps of MAPK **(C)**, photosystem II **(D)**, and HSPs **(E)** related genes in HS and HT varieties under heat stress; **(F)** Confirmation of *BraHSP70-5* gene identified through RNA-seq using RT-qPCR analysis. Asterisks indicate significance between treatment and control using Student’s t-test at **P < 0.01 and ***P < 0.001.

Gene families, including HSPs, cytochrome P450 (CYP), photosystem II (PSII), MAPK, and phenylalanine ammonia-lyase (PAL), play a significant role in plant abiotic and biotic stresses. HSP genes were clustered into two groups: (1) two genes up-regulated at high-stress conditions; (2) initially up-regulated and then down-regulated under high stress (**Figures 5A–F** **;** **Table S3**). Forty-two cytochrome P450 (CYP450) genes were differentially expressed under heat stress, with 16 were up-regulated in both varieties at 1, 6, and 12 h, including *BraCYP98A3*, *BraCYP82G1 BraCYP83A1, BraCYP83A1, BraCYP72A13*, and *BraCYP94B3* (**Figure 5A** **;** **Table S3**). Of 16 DEGs, three genes (*BraCYPBC1*, *BraCYP97B3*, and *BraCYP77A4*) were up-regulated at 1, 6, and 12 h, while the others were up-regulated after 6 and 12 h stress (**Figure 5A**). Furthermore, *BraA01g002420.3C* (*BraCYP84A1-X1*) was down-regulated under heat stress (**Figure 5A**), with similar results obtained using RT-qPCR (**Figure 5B**). Photosynthesis is mainly sensitive to heat stress, and photosystem II (PSII) is the stress-sensitive site with its oxygen-evolving complex. Three PSII genes [*PSBQ3 (BraA02g017000.3C), PSBQ2 (BraA03g024820.3C)*, and *Psb27 (BraA08g035330.3C)*] were negatively expressed in HS (**Figure 5D** **;** **Table S3** **).** Another gene, *PSBQ2 (BraA05g013730.3C)*, encoding PSII oxygen-evolving enhancer protein 2, was up-regulated in HS and HT varieties. Moreover, 5 MAPK and 2 PAL gene expressions significantly differed in HS and HT after 1, 6, and 12 h. PAL1 and PAL3 have a redundant role in flavonoid biosynthesis (**Table S3**). *BraA09g032810.3C* (*MPK-10)* gene was down-regulated, while other genes were up- and down-regulated at different time points (**Figure 5C**).

### Differential expression of transcription factors during heat stress

**Figure 6:**
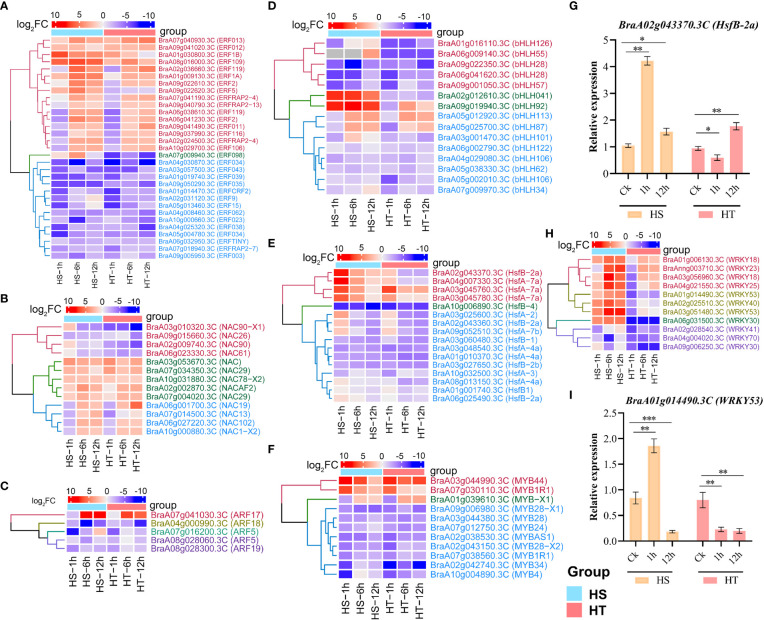
Heatmaps of transcription factor families involved in abiotic stress and validation of DEGs in heat-sensitive (3T-6) and heat-tolerant (Youlu-501) flowering Chinese cabbage using RT-qPCR analysis. The up- and down-regulated genes of TF families: **(A)** ERFs, **(B)** NAC, **(C)** ARFs, **(D)** bHLHs, **(E)** HSFs, **(F)** MYBs, and **(H)** WRKYs for different high-temperature treatments; **(G–I)** Confirmation of *HsfB-2a* and *WRKY53* using RT-qPCR. Asterisks indicate significance between treatment and control using Student’s t-test at *P < 0.01, **P < 0.01, and ***P < 0.001.

TF families of flowering Chinese cabbage were retrieved from the PlantTFDB database for enrichment analysis. We analyzed the DEGs related to heat-responsive TFs, such as MYBs, ARFs, HSFs, NACs, ERFs, and WRKYs families, which could be involved in regulating genes during heat stress (**Figures 6A–I** **;** **Table S4**), identifying 12 MYBs, 13 NACs, 11 WRKYs, 31 ERFs, 17 HSFs, six ARFs, and 16 bHLHs TFs (**Table S4**). Apart from partial bHLH TFs family members, heat stress significantly up-regulated most gene family members of MYBs, NACs, WRKYs, and ERFs. For example, *BraA03g051480.3C (WRKY53)* in HS at 1, 6, and 12 h, with similar results obtained using RT-qPCR (**Figures 6H, I**). *WRKY18* was down-regulated at the initial stress but up-regulated under high-stress conditions in HT (**Figure 6I**). HSFs encoding HSPs, as direct transcriptional activators of genes regulated by heat stress, showed higher up-regulated expression levels in HT than HS: *BraA02g043370.3C* encodes heat stress transcription factor B-2a-like (*HsfB-2a*) and had higher expression levels at 1, 6, and 12 h in HS and HT (**Figures 6F, G** **;** **Table S4**). Similarly, three bZIP TFs (*BZIPHY5*, *BZIPHY5-X1*, and *BZIP61*) were differentially expressed after heat stress. Of 12 MYBs, *BraA07g030110.3C (MYB1R1)* and *BraA03g044990.3C (MYB44)* were up-regulated at 1, 6, and 12 h, with the remaining ten down-regulated (**Figure 6F** **;** **Table S4**). Among 13 NACs, nine genes, including five NACs (*BraA02g002870.3C, BraA03g053670.3C, BraA07g004020.3C, BraA07g034350.3C*, and *BraA10g031880.3C*) were up-regulated in HS and HT at 1, 6, and 12 h, while *BraA06g027220.3C, BraA07g014500.3C*, and *BraA10g000880.3C* were up-regulated under high stress. In addition, most of the auxin (ARFs) and ethylene-responsive transcription factor (ERFs) up-regulated at 12h; for example, e.g., *BraA01g030800.3C, BraA06g041230.3C, BraA09g022610.3C, BraA09g022620.3C*, and *BraA02g031120.3C*, were down-regulated at initial stress (1h) and up-regulated at 6 and 12 h in HS and HT (**Figures 6A, C–I** **;** **Table S4**). These transcription factors possibly involve different thermotolerance in the two genotypes (**Table S4**) and might play essential roles in flowering Chinese cabbage heat resistance during the reproductive stage.

### Validation of RNA-seq data by quantitative real-time PCR

**Figure 7:**
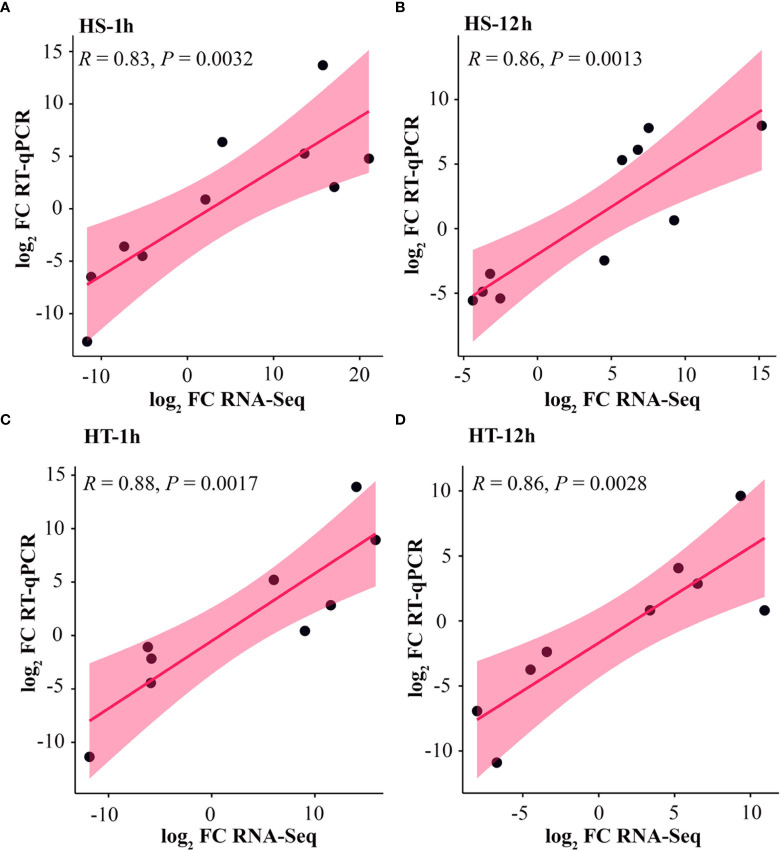
Validation of ten differentially expressed genes in heat-sensitive (3T-6) and heat-tolerant (Youlu-501) flowering Chinese cabbage based on RT-qPCR. The RNA-seq and RT-qPCR values in the form of log_2_ fold-change were plotted using linear regression analysis at **(A)** HS-1, **(B)** HS-12h, **(C)** HT-1h, and **(D)** HT-12h. *R* and *P* indicate the correlation coefficient and corresponding *P*-value.

To verify the results of RNA-seq, ten genes with diverse expression profiles, including up-regulated or down-regulated at all stages and/or regulated in HT or HS, were randomly selected for real-time qPCR to measure expression levels. **Table S5** lists the primer sequences of selected genes. The relative expression level was calculated with 2^−ΔΔCT^ using *BraActin* as a reference gene (**Table S5**). As a result, two TFs genes, *WRKY53* and *HsfB-2a*, had similar expression patterns as those in RNA-seq (**Figure 6G, I** **)**. In brief, all ten genes exhibited the same expression profile, with high correlation coefficients observed between RNA-seq data and RT-qPCR at HS-1h (*r* = 0.83, *P* = 0.0032), HS-12h (*r* = 0.86, *P* = 0.0013), HT-1h (*r* = 0.88, *P* = 0.0017), and HT-12 (*r* = 0.86, *P* = 0.0028), indicating the consistency of RNA sequencing data (**Figure 7**).

## Discussion

Flowering Chinese cabbage, domesticated from Chinese cabbage with flowering stalk, is a major food due to its high nutrient value (Wang et al., 2016). However, heat stress significantly affects the yield of flowering Chinese cabbage. Thus, developing new varieties with high heat resistance is critical for solving this dilemma. In previous studies, stress-associated genes have been reported and functionally annotated in different vegetable crops under biotic and abiotic stress conditions, including high temperature, salt stress, heavy metal stress, drought, nutrient deficiency, bacterial wilt, fungal stress, and UV-B radiation (Gill et al., 2016; Gill et al., 2017; Cai et al., 2020; Yao et al., 2020; Ikram et al., 2022a; Li et al., 2022). In this study, the comparative transcriptome analysis between the heat treatment and control in HS and HT accessions significantly identified 20,680 DEGs (|log_2_(foldchange)|≥2, FDR ≤ 0.001) (**Figures 2**, **3**), and 3,205 putative candidate genes involved in response to heat stress in flowering Chinese cabbage (**Figure 4** **;** **Table S2**).

Heat stress affects flowering Chinese cabbage growth and productivity and produces ROS in plant cells, causing oxidative damage. In response to stress, a plant defense mechanism against oxidative damage activated antioxidant enzymes, including SOD, POD, and CAT, that efficiently scavenge ROS (Wahid et al., 2007; Hasanuzzaman et al., 2013). In this study, SOD, POD, and CAT activities significantly increased under stress conditions compared to the control, more so in the HT genotype than the HS genotype (**Figure 1A**), as reported elsewhere (Wang et al., 2016; Song et al., 2019). Wahid et al. (2007) also measured increasing SOD, POD, and CAT activities in response to stress. Using RNA sequencing, we obtained 21.94–24.90 million raw reads from HS and HT accessions at 0, 1, 6, and 12 h, with 73.06–76.72% of the clean reads mapped to the *B. rapa* reference genome (**Table S1**) and higher from Chinese cabbage leaf RNA sequencing (Zhao et al., 2013) and flowering Chinese cabbage stalk RNA sequencing (Huang et al., 2017). We compared the expression data of leaves at 1, 6, and 12 h with the control, identifying 10,153, 7,879, 7,662, 13,704, 5,161, and 7,438 DEGs in HS-1h, HS-6h, HS-12h, HT-1h, HT-6h, and HT-12h, respectively (**Figures 2A–C**). Further, these genes were involved in cell division, cellular process, response to stimulus, primary metabolic process, response to heat, the developmental process involved in reproduction, photosynthesis, detoxification, RNA binding, and auxin homeostasis (**Figures 3**, **S2**, **S3**) in line with previous studies (Wang et al., 2016; Cai et al., 2020; Helal et al., 2021; Lai et al., 2021). In addition, most DEGs were enriched in the MAPK signaling pathway – plant, brassinosteroid biosynthesis, plant–pathogen interaction, plant hormone signal transduction, and circadian rhythm (**Figures 3**, **S4**, **S5**); these results are similar to previous reports (Song et al., 2014a; Song et al., 2021).

Hierarchical clustering analysis identified three clusters containing 1,958, 591, and 556 down-regulated, up- and down-regulated, and up-regulated, respectively (**Figure 4** **;** **Table S2**), involved in response to stimulus, biological regulation, oxidation, reproduction, cellular process, developmental process, and carbon utilization. These findings indicate that heat stress caused oxidative damage, leading to the up-regulation of genes to increase tolerance; for example, *BraA07g024750.3C* and *BraA10g001200.3C* encoded abscisic acid receptor *PYL9*-like and gibberellin 2-beta-dioxygenase 6, respectively (**Table 1**), and their homologous genes have been functionally characterized to mediate salt stress tolerance in *Arabidopsis* (Hichri et al., 2016) and cassava (Chang et al., 2020). *BraA05g032350.3C* encodes bidirectional sugar transporter SWEET2-like and plays a role in the organic substance biosynthetic process (**Table 1**). Moreover, cluster 3 DEGs were involved in sugar metabolism and proline/polyamine metabolism (**Table S2**), with the accumulation of sugar and proline detected as osmolytes, enhancing heat stress resistance (Wahid et al., 2007; Kumazaki and Suzuki, 2019; Song et al., 2019). The *Sid2-1* mutant was reported in *Arabidopsis*, significantly increasing soluble sugars to improve drought and heat stress (Kumazaki and Suzuki, 2019). Similarly, heat stress increased proline content in leaves of soybean (Amirjani, 2010), tomato (Rivero et al., 2004), chickpea (Chakraborty and Tongden, 2005), and *Arabidopsis* (Zhang and Huang, 2013; Gurrieri et al., 2020; Hoermiller et al., 2022). The cluster 2 DEGs (**Figure 4** **;** **Table S2**) are associated with late embryogenesis abundant protein (Magwanga et al., 2018) and ubiquitin (Qian et al., 2020a), which protect sub-cellular and cellular structures from dehydration and oxidative forces. Therefore, the transcriptomic results were similar to enzymatic activities, and the DEGs related to different proteins in flowering Chinese cabbage might be necessary for heat stress tolerance.

In this study, we identified 42 CYPs DEGs involved in primary and secondary metabolism, including *BraCYP83A1, BraCYP83B1, BraCYP71A1, BraCYP71B13, BraCYP71B2, BraCYP71B37*, and *BraCYP71B9* regulated in HS and HT (**Figure 5** **;** **Table S3**). *BraCYP83A1* was up-regulated at all time points (**Figure 5**), with a similar result reported in *Arabidopsis* that the *CYP83A1* was involved in flavonoid metabolism (Bilodeau et al., 1999). Similarly, *CYP71A1* genes were up-regulated in *Panicum virgatum* under heat stress and responsible for the synthesis of indole alkaloid (Li et al., 2013) and down-regulated in *Rhazya stricta* at high temperatures (Obaid et al., 2016). One gene (*CYP71A23)* in this study was previously identified using genome-wide association study, which caused pollen sterility in *Brassica napus* under heat stress (Rahaman et al., 2018). Similarly, five MAPK genes were identified in this study, which are critical regulators of plant growth in stress responses (Agrawal et al., 2002; Rohila and Yang, 2007). TFs are essential regulators of genes, involving many growth, developmental, and stress processes. The published studies have reported that heat stress increased the ethylene response, auxin response, zing finger proteins, MADS-box proteins, AP2 domain, WRKY, and leucine zipper factors (Chen et al., 2012; Zander et al., 2012; Kim et al., 2016; Wang et al., 2016; Gu et al., 2018; Rehman et al., 2021). RNA-seq analysis of HS and HT lines in rice and maize determined ethylene response factors that increased stress tolerance (Thirunavukkarasu et al., 2013; Minami et al., 2018). In this study, 31 ERFs were enriched, with >12 up-regulated at 6 and 12 h, possibly playing a significant role in heat tolerance (**Figure 6A**). Likewise, five WRKYs (*BraWRKY18, BraWRKY53, BraWRKY23, BraWRKY25*, and *BraWRKY40*) were up-regulated in HS and HT at 6 and 12 h (**Figure 5** **;** **Table S4**). Of these, *WRKY25* was also reported for heat tolerance in *Arabidopsis* (Chen et al., 2012), and *WRKY26* and *WRKY33* were also reported for stress resistance (Chen et al., 2012; Kim et al., 2016; Gu et al., 2018). Moreover, many other TFs, such as MYB, NAC, bHLH, and ARF, were detected (**Figure 6** **;** **Table S4**), indicating that TF families are directly related and enhance heat stress tolerance in flowering Chinese cabbage.

HSPs are expressed more during the initial stage of stress than long-term stress (Singh et al., 2019; Qian et al., 2020b). In this study, only ten DEGs were annotated as HSPs, including *BraHSP70-5, BraHSP70-10, BraHSP70-8*, and *BraHSP70-6* up-regulated at 1h and *BraHSP70-6-90-1* and *BraHSP70-17* up-regulated at 12h (**Figure 5** **;** **Table S3**). Likewise, Wang et al. (2016) reported that *HSP70* and *HSPB27* were expressed in *B. rapa* after heat stress, with *HSP70* negatively controlling the heat stress response. *HSP70s* have been identified in many vegetables in response to heat stress, such as potato, tomato, cabbage, and pepper (Guo et al., 2016; Lee et al., 2017; Liu et al., 2018). Studies have demonstrated that HSFs are key regulators of HSPs under heat stress (Wang et al., 2016; Liu et al., 2018), with 35 HSF genes in A, B, and C groups reported in Chinese cabbage (Song et al., 2014b). However, 17 BraHSFs genes identified in this study, including *HsfB1, HsfB-2a, HsfA-7a, HsfB-1, HsfA-4a, HsfA-7b)*, and *HsfA-3*, were up-regulated at 1, 6, and 12 h in HS and HT (**Figure 6** **;** **Table S4**). Similarly, Scharf et al. (2012) identified *HSFA2* as a major heat stress factor that induces HSPs expression in stressed plants. Moreover, overexpression of *HSFA2* significantly increased salt, heat, light, and drought stress tolerance (Guo et al., 2016). A tomato *HSFA1-a* was identified as a master regulator for heat response in rice (Mishra et al., 2018). Thus, the present study increased our understanding of DEGs, their roles in stress, and *BraHSF* genes under heat stress.

## Conclusion

We compared the transcriptomes of HS and HT varieties of flowering Chinese cabbage under heat stress. Approximately 3,205 genes were differentially expressed, with 1,078 DEGs identified at 1, 6, and 12 h after heat stress in both varieties. Cluster analysis divided these genes into three clusters containing 1,958, 591, and 556 genes, which participated in response to stimulus, cell division, cellular process, heat, programmed cell death, ribosome biogenesis, etc. Finally, 15 potential heat-tolerant genes were identified based on functional annotation and literature search. These results provide useful genetic resources for understanding the heat-tolerance mechanism in flowering Chinese cabbage, and these candidate genes require further functional validation and cloning to determine their actual role in heat tolerance.

## Data availability statement

The original contributions presented in the study are publicly available. This data can be found here: NCBI, PRJNA885253.

## Author contributions

PG and YX conceived and designed the experiments. JC, YX, MI, and RL performed the experiments and analyzed data. YX, MI, and RL contributed reagents/materials/analysis tools. MI, JC, YX, RL, KS, and PG wrote the paper. All authors contributed to the article and approved the submitted version.

## Funding

This work was funded by the Natural Science Foundation of Guangdong Province, China (2019A1515011587).

## Acknowledgments

We thank the Guangdong Academy of Agricultural Sciences, China, for providing flowering Chinese cabbage accessions.

## Conflict of interest

The authors declare that the research was conducted in the absence of any commercial or financial relationships that could be construed as a potential conflict of interest.

## Publisher’s note

All claims expressed in this article are solely those of the authors and do not necessarily represent those of their affiliated organizations, or those of the publisher, the editors and the reviewers. Any product that may be evaluated in this article, or claim that may be made by its manufacturer, is not guaranteed or endorsed by the publisher.
