# Supplementary material for: Comprehensive transcriptome analysis reveals heat-responsive genes in flowering Chinese cabbage (Brassica campestris L. ssp. chinensis) using RNA sequencing
Source: Front Plant Sci. 2022 Dec 2;13:1077920. doi: 10.3389/fpls.2022.1077920 (PMC9755508; doi:10.3389/fpls.2022.1077920)
Supplement: Supplementary file 2 [file Presentation_1.pdf]

Supplementary

Comprehensive transcriptome analysis reveals heat-responsive genes in flowering Chinese cabbage (*Brassica campestris* L. ssp. *chinensis*) using RNA sequencing

Muhammad Ikram[1, †], Jingfang Chen[1, †], Yanshi Xia[1,*], Ronghua Li[1], Kadambot H.M. Siddique[2], Peiguo Guo[1,*]

[1] Guangdong Provincial Key Laboratory of Plant Adaptation and Molecular Design, International Crop Research Center for Stress Resistance, School of Life Sciences, Guangzhou University, Guangzhou 510006, China

[2] The UWA Institute of Agriculture, UWA School of Agriculture & Environment, The University of Western Australia, Perth, WA 6001, Australia

* Corresponding author: guopg@gzhu.edu.cn;

xiayanshi922@163.com

† Authors contributed equally to this work

Figures S1-S5

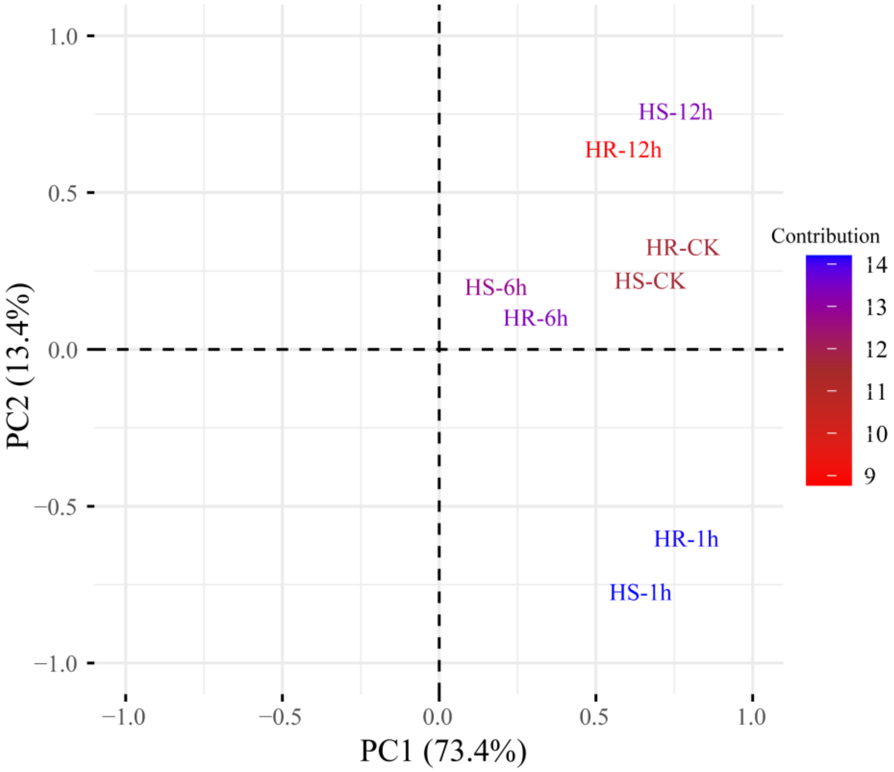

Figure S1. Principal component analysis using FPKM values of heat-sensitive and heat-tolerant flowering Chinese cabbage varieties.

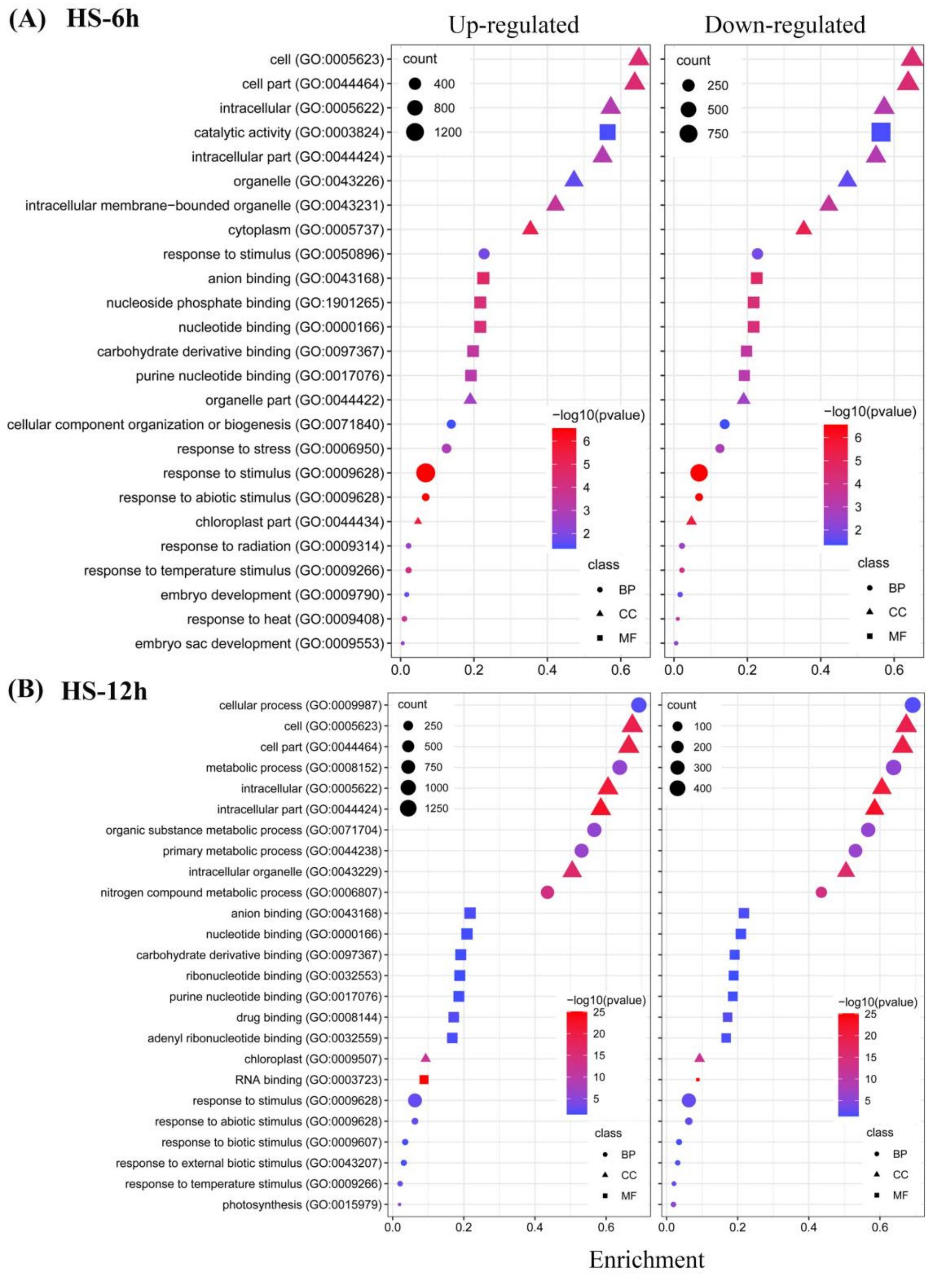

Figure S2. GO enrichment analysis of up- and down-regulated genes in heat-sensitive (3T-6) flowering Chinese cabbage. The top 25 GO terms in three categories are listed as (**A**) HS-CK vs. HS-6h and (**B**) HS-CK vs. HS-12h at $P \leq 0.05$.

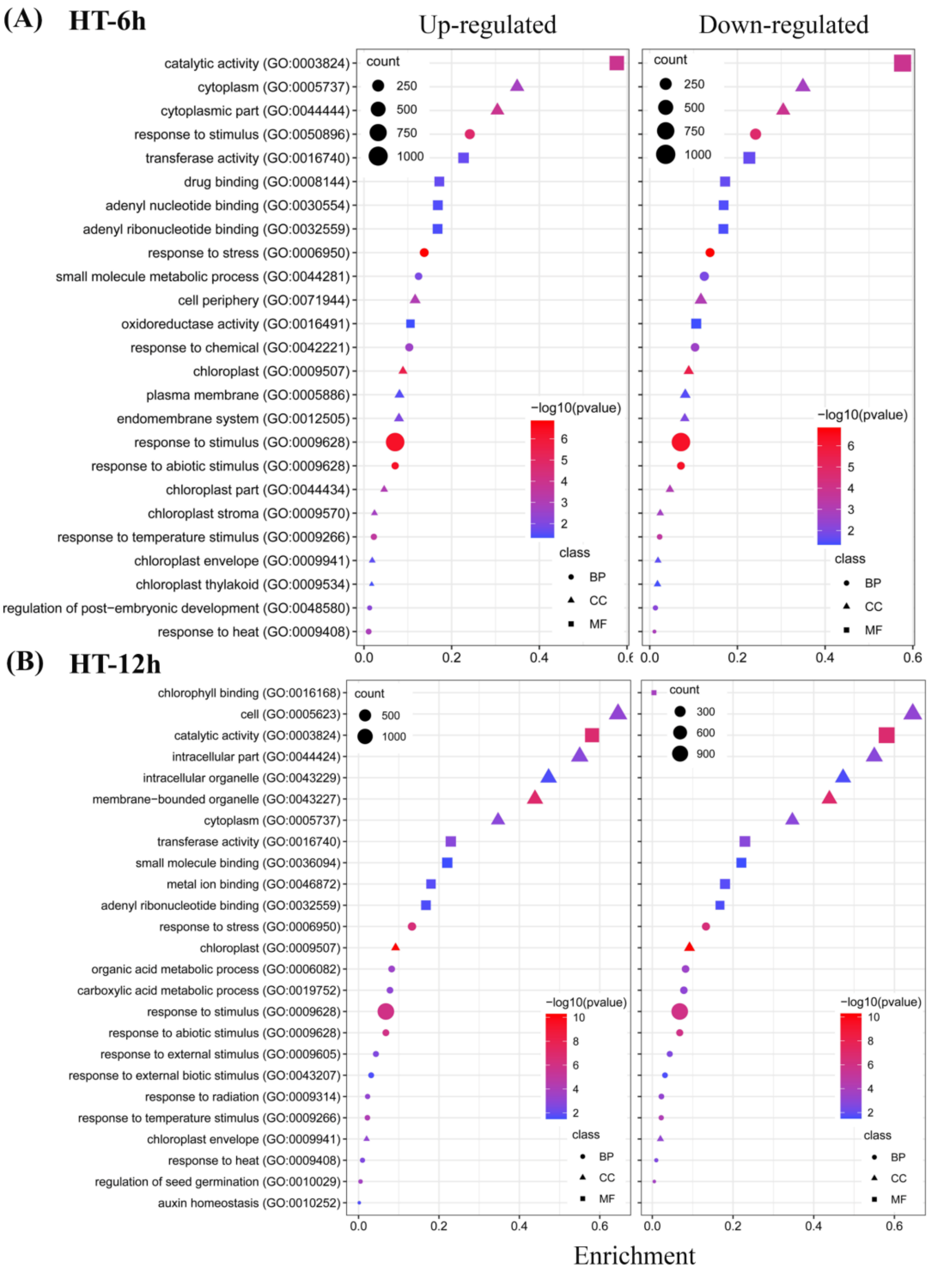

Figure S3. GO enrichment analysis of up- and down-regulated genes of heat-tolerant (Youlu-501) flowering Chinese cabbage. The top 25 GO terms in three categories are listed as (**A**) HT-CK vs. HT-6h and (**B**) HT-CK vs. HT-12h at $P \leq 0.05$.

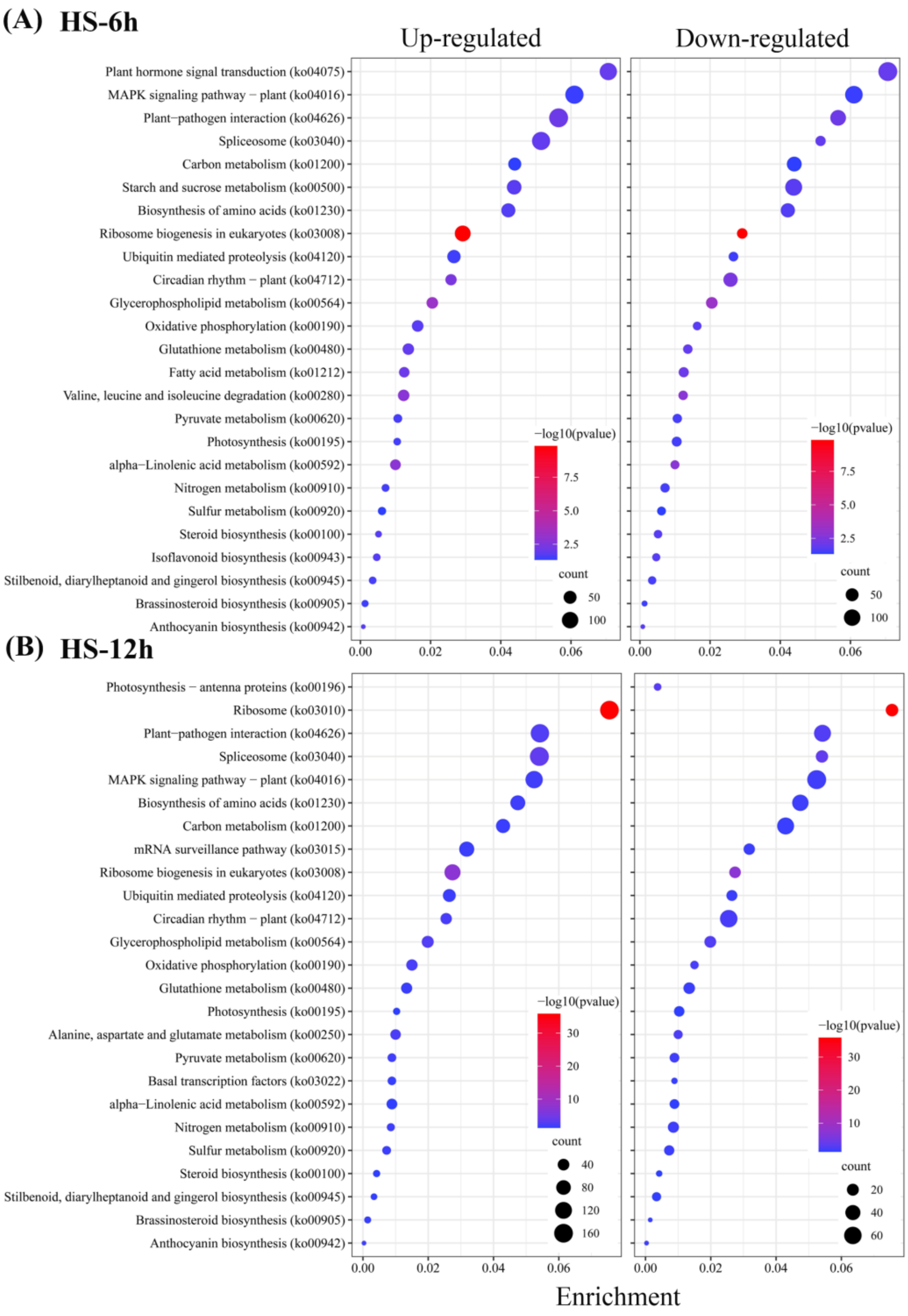

Figure S4. KEGG enrichment analysis of up- and down-regulated genes of heat-sensitive (3T-6) flowering Chinese cabbage. The top 25 pathways are listed as (**A**) HS-CK vs. HS-6h and (**B**) HS-CK vs. HS-12h at $P \leq 0.05$.

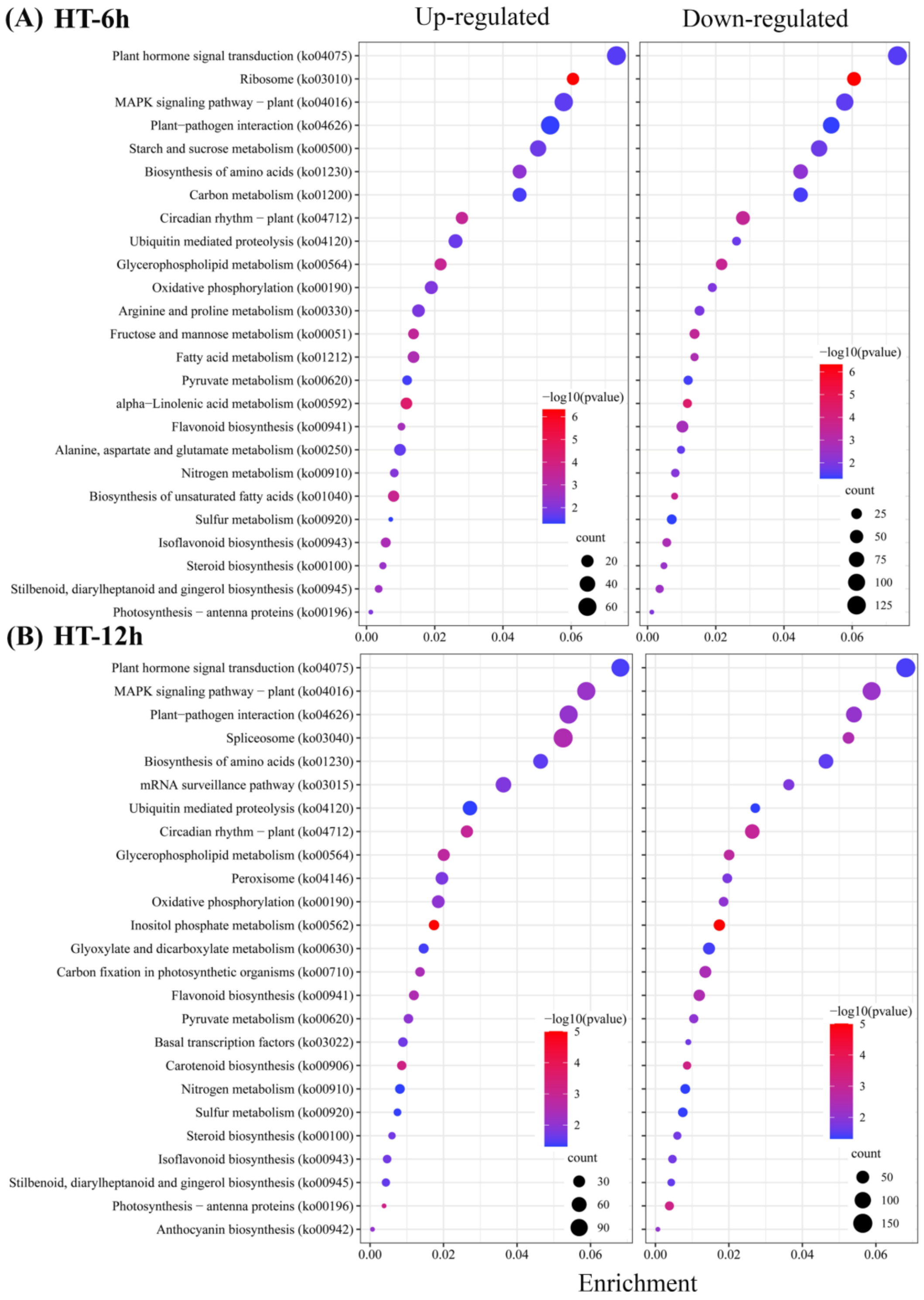

Figure S5. KEGG enrichment analysis of up- and down-regulated genes of heat-tolerant (Youlu-501) flowering Chinese cabbage. The top 25 pathways are listed as (**A**) HT-CK vs. HT-6h and (**B**) HT-CK vs. HT-12h at $P \leq 0.05$.
